# Early-Onset Fetal Growth Restriction Increases Left Ventricular Sphericity in Adolescents Born Very Preterm

**DOI:** 10.1007/s00246-023-03265-z

**Published:** 2023-08-19

**Authors:** Jonas Liefke, Alvaro Sepúlveda-Martinez, Snehlata Shakya, Katarina Steding Ehrenborg, Håkan Arheden, Eva Morsing, David Ley, Einar Heiberg, Erik Hedström

**Affiliations:** 1 Clinical Physiology, Department of Clinical Sciences Lund, Lund University, Lund, Sweden; 2 Department of Clinical Physiology, Skåne University Hospital, Lund, Sweden; 3 Fetal Medicine Unit, Department of Obstetrics and Gynecology, Hospital Clínico de La Universidad de Chile, Santiago de Chile, Chile; 4 Paediatrics, Department of Clinical Sciences Lund, Lund University, Lund, Sweden; 5 Department of Pediatrics, Skåne University Hospital, Lund, Sweden; 6 Wallenberg Center for Molecular Medicine, Lund University, Lund, Sweden; 7 Diagnostic Radiology, Department of Clinical Sciences Lund, Lund University, Lund, Sweden; 8 Department of Radiology, Skåne University Hospital, Lund, Sweden

**Keywords:** Cardiac remodeling, Shape analysis, Fetal growth restriction, Preterm delivery

## Abstract

Left ventricular shape alterations predict cardiovascular outcomes and have been observed in children born preterm and after fetal growth restriction (FGR). The aim was to investigate whether left ventricular shape is altered in adolescents born very preterm and if FGR has an additive effect. Adolescents born very preterm due to verified early-onset FGR and two control groups with birthweight appropriate for gestational age (AGA), born at similar gestational age and at term, respectively, underwent cardiac MRI. Principal component analysis was applied to find the modes of variation best explaining shape variability for end-diastole, end-systole, and for the combination of both, the latter indicative of function. Seventy adolescents were included (13–16 years; 49% males). Sphericity was increased for preterm FGR versus term AGA for end-diastole (36[0–60] vs − 42[− 82–8]; *p* = 0.01) and the combined analysis (27[− 23–94] vs − 51[− 119–11]; *p* = 0.01), as well as for preterm AGA versus term AGA for end-diastole (30[− 56–115] vs − 42[− 82–8]; *p* = 0.04), for end-systole (57[− 29–89] vs − 30[− 79–34]; *p* = 0.03), and the combined analysis (44[− 50–145] vs − 51[− 119–11]; *p* = 0.02). No group differences were observed for left ventricular mass or ejection fraction (all *p* ≥ 0.33). Sphericity was increased after very preterm birth and exacerbated by early-onset FGR, indicating an additive effect to that of very preterm birth on left ventricular remodeling. Increased sphericity may be a prognostic biomarker of future cardiovascular disease in this cohort that as of yet shows no signs of cardiac dysfunction using standard clinical measurements.

## Introduction

Pregnancies complicated by preeclampsia, fetal growth restriction (FGR), and preterm birth may result in low birth weight [1]. Low birth weight and prematurity in itself induce cardiometabolic programming which may result in development of diabetes, hypertension, and increased cardiovascular mortality [2–9]. To what extent verified FGR further increases long-term cardiovascular risk related to preterm delivery is not known.

A possible window of opportunity for preventive care may still exist in childhood [10, 11] and adolescence [12, 13]. However, routine clinical biomarkers of cardiac remodeling, such as left ventricular (LV) volume, ejection fraction, and LV hypertrophy, may not be sensitive enough to detect early signs of future disease [14]. Left ventricular sphericity is an indicator of cardiac remodeling and is a predictive biomarker of future cardiovascular disease, potentially more so than clinical biomarkers of cardiac remodeling [15, 16]. Shape analysis based on cardiovascular magnetic resonance (CMR) imaging has indicated both left and right ventricular remodeling after preterm birth [17, 18]. However, it is unclear whether verified FGR adds to shape changes related to preterm birth, and thereby further increase risk of future cardiovascular disease.

The aims of this study were therefore to investigate (1) whether left ventricular end-diastolic, end-systolic, and combined end-diastolic and end-systolic shapes differ in adolescents born very preterm with or without verified preceding early-onset FGR compared to healthy controls; and (2) whether verified early-onset FGR exacerbates left ventricular shape changes due to very preterm birth.

## Methods

### Study Population and Design

The regional Ethics review board approved the study (DNR 2013/244) and all participants and their guardians as appropriate provided written informed consent before participation. This prospective follow-up cohort study was conducted at Skåne University Hospital and participants underwent CMR imaging between 2014 and 2019.

Participants were identified from a cohort delivered between 1998 and 2004 at the Department of Obstetrics and Gynaecology in Lund and the study consists of adolescents born very preterm with or without verified preceding early-onset FGR and term controls [19]. Exclusion criteria were malformation, chromosomal aberration, and twin-to-twin transfusion. The original cohort consisted of three groups with 34 fetuses each; the preterm FGR group with the following inclusion criteria: estimated fetal weight more than 2 SD below the mean of the Swedish reference population [20] and FGR verified by absent or reversed end-diastolic flow in the umbilical artery, delivered on fetal indication before 30 weeks of gestation; the control group preterm AGA with birth weight appropriate for gestational age (AGA) matched for sex, gestational age and year of birth; and the control group term AGA matched for sex and year of birth. Perinatal outcome and follow-up studies on cardiovascular, pulmonary, and neuro-cognitive outcomes in childhood [19, 21, 22] and renal and cardiovascular outcome in adolescence [12, 13] have previously been described. All individuals, now in adolescence, from the original above groups were asked to participate in the current study.

### CMR Imaging

Participants underwent CMR in supine position using a 1.5 T Philips Achieva (Best, the Netherlands) or Siemens Aera (Erlangen, Germany) MR scanner. For quantification of LV volumes and LV shape, cine images were acquired in short-axis and 2-, 3-, and 4-chamber long-axis views. Cine images were acquired using a balanced steady-state free precession (bSSFP) sequence with typical parameters 1.2 × 1.2 × 6 mm, slice gap 2 mm, flip angle 51°, TR 73 ms, TE 1.2 ms.

Height, weight, and resting blood pressure were assessed in conjunction with CMR. Body surface area (BSA) was calculated using the Mosteller formula [23].

### CMR Image Analysis

#### Left Ventricular Volumes and Function

Images were analyzed using the freely available software Segment version 3.0 (Medviso AB, Lund, Sweden) [24]. Left ventricular volumes were measured in end-diastole and end-systole by manual delineation of the endo- and epicardium in short-axis cine images covering the entire left ventricle [25], guided by long-axis views. Left ventricular mass (LVM) was calculated as the ventricular wall volume multiplied by left ventricular myocardial density (1.05 g/ml).

#### Shape Analysis

The above delineations were used as input for shape analysis. Right ventricular insertion points were manually annotated in the entire short-axis stack and used for aligning the outflow tract and the LV center of gravity. Any misalignment between slices was corrected for using 3D center of gravity curve fitting.

The delineations in each slice were stored as 80 coordinate datapoints, respectively. Each individual cardiac stack was resampled to 20 slices, whereby the cardiac stack for one subject included information from 3200 points (80 coordinate datapoints × 2 time points × 20 slices). All study subjects from the three separate groups were initially combined and a total of 70 subjects × 3200 points were used for analysis. Principle component analysis (PCA) was applied to find the modes of variation that best explain the shape variability within the whole dataset. Analysis was performed unadjusted, adjusted for BSA, and adjusted for end-diastolic volume (EDV). The most explanatory shape modes were computed for end-diastole, end-systole, and for the combination of both, where the combined analysis yields the shape change between end-diastole and end-systole, thus including contraction in the shape analysis. The seven modes that best explained the shape variance were selected for further analysis. Left ventricular shapes were presented unadjusted and adjusted for potential confounders between groups.

The modes with highest variance for the three groups combined were separated for visualization as 3D surfaces for end-diastole, end-systole, and for the combination of both, whereafter shape data were separated to enable statistical analyses between groups. A traditional shape parameter was ascribed to the respective mode when the mode and the shape parameter were visually congruent. This was not possible for all the modes. Traditional shape parameters are length, width, sphericity (i.e., the ratio of maximum short-axis diameter to length), wall thickness, conicity (i.e., ratio of 2nd apical diameter to middle slice diameter in the short-axis views), curvedness, apex orientation, basal area and basal area orientation. The purpose of the comparison between shape modes and traditional shape parameters was to ascribe each mode a textual description that allows for visual interpretation of the shape modes.

### Statistical Analyses

Statistical analyses were performed using SPSS 26.0 (IBM Corp, Armonk, New York, USA) and GraphPad Prism 9.02 (GraphPad Software, La Jolla, California, USA). Distribution of continuous variables was assessed through histogram visualization. Data are expressed as median (range). The Kruskal–Wallis test assessed group differences with Dunn’s or Bonferroni’s multiple comparison test, as appropriate. Pearson’s chi square test or Fisher’s exact test was used for categorical variables. *P* < 0.05 were considered to show statistically significant differences.

## Results

### Study Population

Of the 102 potential participants from the original cohort, all were contacted and 71 accepted to participate in the current study. One individual aborted the CMR examination and was excluded. Table 1 describes the characteristics at birth and adolescence of the 70 adolescents included for analyses.

**Table 1:** Characteristics perinatally and in adolescence

	Preterm FGR	Preterm AGA	Term AGA	*P*			
				Between all groups	Preterm FGR vs Preterm AGA	Preterm FGR vs Term AGA	Preterm AGA vs Term AGA
	*n* = *22*	*n* = *22*	*n* = *26*				
*Characteristics perinatally*							
Gestational age at birth (weeks + days)	26 + 6 (24 + 4–29 + 1)	27 + 2 (24 + 3–29 + 5)	40 + 0 (38 + 3–40 + 5)	**< 0.0001**	1	** < 0.0001**	** < 0.0001**
Birthweight (g)	655 (395–976)	1,060 (660–1,790)	3,485 (3,000–4,390)	** < 0.0001**	**0.01**	**< 0.0001**	** < 0.0001**
Birthweight deviation (%)	−35 (−63 to −22)	−3 (−23 to 14)	−2 (−17 to 21)	** < 0.0001**	** < 0.0001**	** < 0.0001**	1
Blood flow class	IIIA/IIIB	N/A	N/A				
Maternal preeclampsia	8 (36)	1	0				
Prenatal steroid n (%)	22 (100)	21 (95)^1^	0				
Cesarean section n (%)	22 (100)	12 (55)	0		**0.0001**		
*Characteristics in adolescence*							
Age (years)	15 (13–16)	15 (13–16)	15 (13–16)	0.85			
Girls [*n* (%)]	12 (55)	10 (45)	14 (54)	0.8			
Weight (kg)	51 (38–90)	57 (37–74)	58 (37–89)	0.3			
Height (cm)	160 (150–180)^*^	169 (149–183)	167 (155–189)	**0.02**	**0.04**	**0.04**	1
BSA (m^2^)	1.5 (1.3–2.1)	1.6 (1.2–1.9)	1.7 (1.3–2.2)	0.13			
SBP (mmHg)	105 (87–123)	107 (88–120)	102 (89–130)	0.37			
DBP (mmHg)	53 (41–74)	54 (45–65)	50 (44–80)	0.08			

Bold values indicate significant differences

*FGR* fetal growth restriction, *AGA* appropriate for gestational age, *SBP* systolic blood pressure, *DBP* diastolic blood pressure

^*^Girls born preterm FGR were shorter than term AGA (158 cm (150–165 cm) vs 164 cm (157–176 cm); *p* = 0.006)

^1^One data point missing

### CMR Image Analysis

#### Left Ventricular Volumes and Function

Left ventricular end-diastolic (80 vs 88 ml/m^2^; *p* = 0.02) and end-systolic (34 vs 41 ml/m^2^; *p* = 0.02) volumes indexed for BSA were smaller for the preterm AGA group as compared to the term AGA group (Table 2). There were no differences in left ventricular mass or measures of left ventricular function between groups (all *p* ≥ 0.1) (Table 2).

**Table 2:** Cardiac volumes and function by CMR

	Preterm FGR	Preterm AGA	Term AGA	*P*			
	*n* = *22*	*n* = *22*	*n* = *26*	Between all groups	Preterm FGR vs Preterm AGA	Preterm FGR vs Term AGA	Preterm AGA vs Term AGA
Left ventricular mass (g)	70 (42–113)	71 (42–112)	76 (50–147)	0.48			
Left ventricular mass/BSA (g/m^2^)	47 (32–64)	43 (25–70)	47 (33–75)	0.22			
End-diastolic volume (ml)	122 (74–201)	128 (89–197)	141 (94–213)	0.11			
End-diastolic volume/BSA (ml/m^2^)	82 (57–113)	80 (47–116)	88 (62–110)	**0.02**	1	0.26	**0.02**
End-systolic volume (ml)	56 (33–101)	52 (37–92)	64 (37–97)	**0.049**	1	0.39	**0.049**
End-systolic volume/BSA (ml/m^2^)	36 (25–55)	34 (23–54)	41 (24–50)	**0.03**	0.37	0.88	**0.02**
Heart rate (bpm)	76 (44–93)	75 (48–128)	76 (57–99)	0.76			
Stroke volume (ml)	68 (41–104)	70 (46–105)	78 (54–117)	0.12			
Ejection fraction (%)	55 (46–62)	56 (51–69)	54 (46–62)	0.33			
Cardiac output (l/min)	4.9 (2.4–7.9)	5.1 (2.8–7.8)	5.4 (3.7–9.2)	0.50			
Cardiac index (l/min/m^2^)	3.3 (1.6–4.6)	3.1 (1.7–4.4)	3.2 (2.4–5.2)	0.85			

Bold values indicate significant differences

*BSA* body surface area, *FGR* fetal growth restriction, *AGA* appropriate for gestational age

#### Unadjusted Shape

**Fig. 1:**
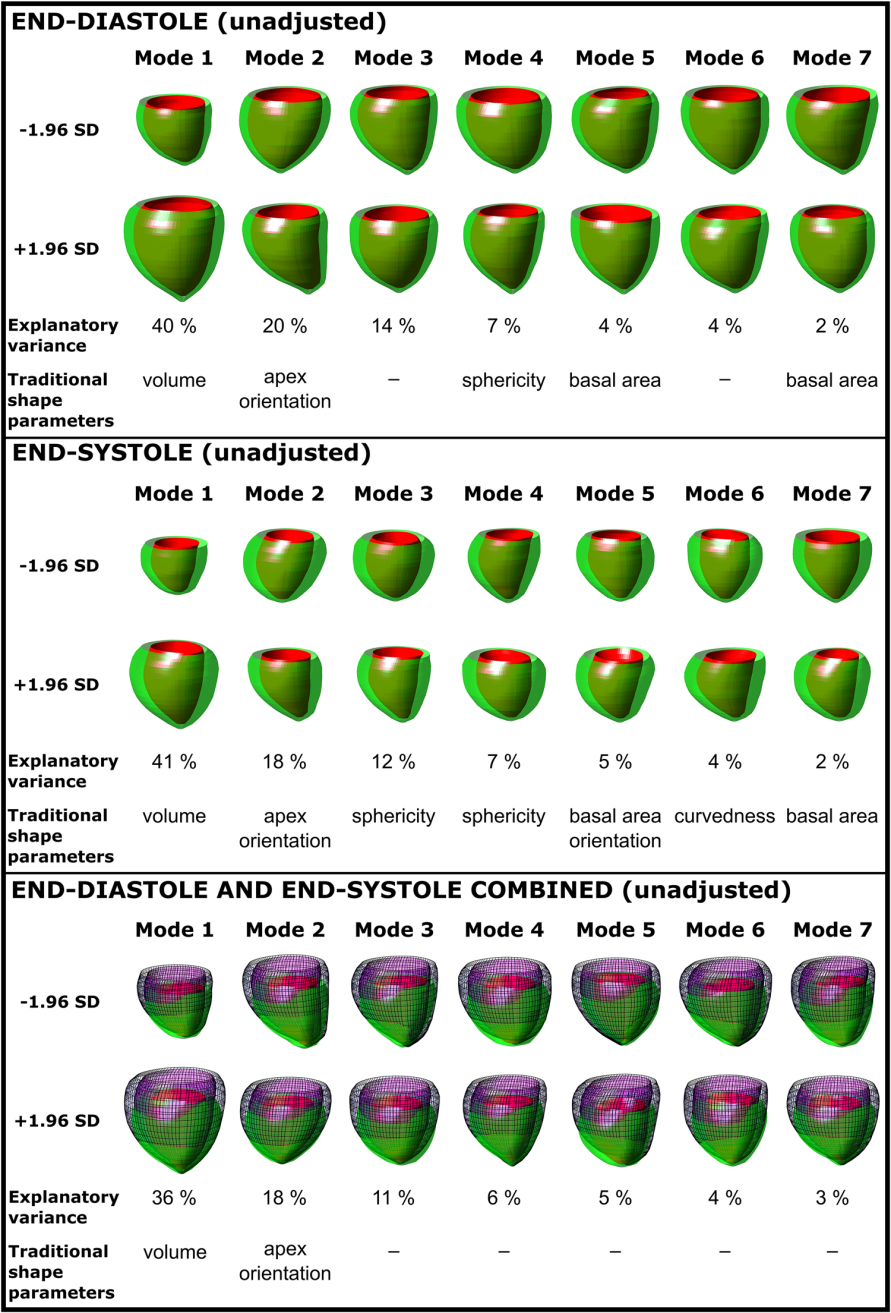
Unadjusted modes 1–7 and their respective shape explanatory variance for end-diastole (top panel), end-systole (middle panel), and end-diastole and end-systole combined (bottom panel). 3D shapes shown are the ± 1.96 standard deviation extremes for each mode. The unadjusted shape analysis captures the major explanatory variances without factoring in body surface area or cardiac size

Figure 1 shows the seven most important shape modes for the whole dataset. The first seven modes explained 91% of end-diastolic, 89% of end-systolic and 83% of combined end-diastolic and end-systolic shape variability. Left ventricular volume (mode 1) was the major contributor to shape variance and explained 40% of the variance within the dataset at end-diastole, 41% at end-systole and 36% in the combined analysis. Table 3 shows the difference in shape modes between groups. Left ventricular volume was smaller in the preterm FGR group as compared to the term AGA group for end-diastolic (− 81 vs 58; *p* = 0.01) and for the combined end-diastolic and end-systolic shape variability (− 84 vs 102; *p* = 0.02). No other modes differed between groups (all *p* ≥ 0.05).

**Table 3:** Shape analysis data for unadjusted, body surface area (BSA) adjusted, and end-diastolic volume (EDV) adjusted modes

	Explanatory variance (%)	Preterm FGR	Preterm AGA	Term AGA	P between groups
		*(n* = *22)*	*(n* = *22)*	*(n* = *26)*	
**Undajusted modes**					
*End-diastole*					
Mode 1	40	−81 (−343 to 251)*	−20 (−237 to 296)	58 (−266 to 366)	**0.011**
Mode 2	20	−22 (−192 to 155)	−10 (−271 to 230)	−14 (−261 to 224)	0.813
Mode 3	14	10 (−148 to 130)	3 (−361 to 220)	23 (−228 to 132)	0.388
Mode 4	7	−9 (−117 to 74)	−9 (−236 to 121)	14 (−88 to 126)	0.278
Mode 5	4	14 (−91 to 114)	−23 (−109 to 66)	17 (−135 to 90)	0.067
Mode 6	4	16 (−90 to 85)	6 (−71 to 117)	−11 (−121 to 71)	0.334
Mode 7	2	−7 (−73 to 53)	6 (−48 to 60)	6 (−93 to 65)	0.285
*End-systole*					
Mode 1	41	−43 (−367 to 278)	−58 (−226 to 299)	66 (−209 to 305)	**0.04**
Mode 2	18	7 (−185 to 198)	6 (−292 to 131)	16 (−212 to 152)	0.795
Mode 3	12	−8 (−138 to 67)	22 (−177 to 223)	−8 (−136 to 102)	0.052
Mode 4	7	−11 (−129 to 120)	5 (−80 to 177)	1 (−119 to 89)	0.742
Mode 5	5	5 (−60 to 75)	3 (−135 to 118)	−1 (−121 to 87)	0.9
Mode 6	4	9 (−62 to 77)	6 (−112 to 76)	−6 (−88 to 102)	0.375
Mode 7	2	−3 (−84 to 79)	8 (−54 to 84)	−1 (−67 to 44)	0.259
*End-diastole end-systole combined*					
Mode 1	36	−84 (−499 to 333)*	−30 (−303 to 373)	102 (−314 to 501)	**0.022**
Mode 2	18	22 (−262 to 290)	13 (−330 to 341)	32 (−308 to 299)	0.964
Mode 3	11	15 (−158 to 184)	−2 (−371 to 285)	33 (−286 to 152)	0.233
Mode 4	6	4 (−152 to 111)	−15 (−360 to 70)	33 (−80 to 160)	0.089
Mode 5	5	4 (−147 to 141)	11 (−86 to 120)	0 (−192 to 109)	0.473
Mode 6	4	−24 (−211 to 101)	11 (−90 to 169)	−5 (−106 to 160)	0.66
Mode 7	3	17 (−92 to 90)	−18 (99 to 105)	−5 (−105 to 154)	0.345
**BSA-adjusted modes**					
*End-diastole*					
Mode 1	31	−35 (−148 to 92)*	−30 (−167 to 256)*	42 (−247 to 325)	**0.008**
Mode 2	24	6 (−162 to 165)	21 (−186 to 204)	−26 (−199 to 173)	0.242
Mode 3	16	0 (−141 to 119)	−8 (301 to 133)	24 (−118 to 103)	0.144
Mode 4	6	0 (−92 to 57)	2 (−89 to 71)	5 (−107 to 93)	0.786
Mode 5	5	13 (−79 to 82)	−16 (−85 to 53)*	15 (−107 to 77)	**0.028**
Mode 6	5	3 (−73 to 74)	−1 (−75 to 92)	−6 (−84 to 72)	0.506
Mode 7	3	17 (−33 to 57)	−7 (−58 to 45)	−3 (−64 to 53)	0.129
*End-systole*					
Mode 1	34	−18 (−237 to 133)	−57 (−153 to 255)*	30 (−171 to 332)	**0.032**
Mode 2	19	−7 (−172 to 124)	8 (−102 to 160)	4 (−110 to 115)	0.789
Mode 3	12	−15 (−105 to 58)**	27 (−121 to 155)	−7 (−141 to 54)	**0.028**
Mode 4	7	3 (−106 to 97)	5 (−113 to 46)	−13 (−70 to 111)	0.452
Mode 5	6	2 (−73 to 51)	4 (−103 to 92)	3 (−102 to 82)	0.955
Mode 6	5	6 (−41 to 66)	−2 (−93 to 62)	−2 (−65 to 60)	0.388
Mode 7	3	−4 (−67 to 58)	5 (−44 to 71)	−4 (−53 to 38)	0.297
*End-diastole end-systole combined*					
Mode 1	26	−27 (−286 to 116)*	−44 (−237 to 327)*	51 (−294 to 436)	**0.006**
Mode 2	21	−26 (−268 to 221)	1 (−227 to 212)	23 (−256 to 246)	0.375
Mode 3	12	11 (−148 to 176)	−22 (−322 to 171)	32 (−152 to 128)	0.083
Mode 4	6	4 (−98 to 89)	−9 (−171 to 112)	18 (−148 to 125)	0.924
Mode 5	6	20 (−130 to 112)	−5 (−128 to 56)	20 (−84 to 88)	0.137
Mode 6	5	−7 (−115 to 93)	−5 (−82 to 165)	6 (−87 to 101)	0.648
Mode 7	3	9 (−99 to 72)	−6 (−96 to 52)	−11 (−76 to 93)	0.429
**EDV-adjusted modes**					
*End-diastole*					
Mode 1	31	0.1 (−3.9 to 3.9)	0.0 (−5.4 to 4.9)	−0.6 (−5.0 to 4.5)	0.586
Mode 2	23	0.50 (−2.5 to 2.7)	0.2 (−6.4 to 5.0)	−0.2 (−5.2 to 3.1)	0.545
Mode 3	16	−0.3 (−3.3 to 2.0)*	−0.3 (−4.3 to 2.3)	0.5 (−2.1 to 3.8)	**0.013**
Mode 4	6	0 (−1.4 to 2.3)	−0.4 (−1.5 to 2.21)	−0.1 (−1.4 to 1.6)	0.483
Mode 5	6	−0.2 (−2.0 to 1.9)	0.30 (−1.0 to 2.1)*	−0.3 (−1.7 to 2.3)	**0.027**
Mode 6	4	0.3 (−0.9 to 1.3)	0 (−1.4 to 1.2)	−0.3 (−1.4 to 2.0)	0.13
Mode 7	3	−0.1 (−2 to 1.0)	0.1 (−1.6 to 1.0)	0.1 (−1.3 to 1.0)	0.421
*End-systole*					
Mode 1	27	−0.1 (−4.6 to 3.8)	−0.4 (−5.3 to 2.5)	0.3 (−3.2 to 4.3)	0.405
Mode 2	21	0.1 (−3.9 to 2.8)	0 (−3.5 to 4.8)	−0.2 (−2.0 to 2.2)	0.725
Mode 3	14	−0.3 (−2.5 to 3.0)	0.8 (−3.3 to 2.9)	−0.3 (−2.3 to 1.6)	0.059
Mode 4	8	0.1 (−1.9 to 1.3)	0.1 (−2.5 to 2.7)	0.2 (−2.4 to 1.7)	0.943
Mode 5	6	0.2 (−2.0 to 2.2)	0.2 (−2.1 to 1.5)	−0.4 (−2.2 to 1.6)	0.216
Mode 6	4	−0.1 (−0.7 to 2.2)	0 (−1.3 to 1.3)	0 (−1.7 to 1.0)	0.653
Mode 7	4	−0.3 (−1.7 to 1.4)	0.2 (−0.8 to 1.7)	0.0 (−1.0 to 1.0)	0.224
*End-diastole end-systole combined*					
Mode 1	25	−0.3 (−5.7 to 5.6)	0.1 (−6.8 to 6.6)	0.6 (−6.1 to 5.8)	0.73
Mode 2	18	0.6 (−3.2 to 4.3)	0.3 (−7.6 to 6.8)	−0.7 (−6.1 to 3.7)	0.189
Mode 3	12	−0.5 (−4.9 to 3.3)	−0.3 (−6.3 to 3.0)*	0.8 (−1.6 to 4.6)	**0.014**
Mode 4	7	0.1 (−3.0 to 3.2)	0.2 (−1.2 to 2.3)	0 (−3.6 to 2.1)	0.399
Mode 5	6	0.1 (−4.0 to 2.3)	0.0 (−2.2 to 4.0)	−0.1 (−2.7 to 2.0)	0.929
Mode 6	5	0 (−2.2 to 2.1)	−0.1 (−2.0 to 2.5)	0.2 (−1.9 to 2.6)	0.707
Mode 7	4	0.7 (−1.5 to 1.9)*	0 (−3.3 to 2.4)	−0.3 (−2.2 to 1.1)	**0.017**

Bold values indicate significant differences

*Compared to term AGA. **Compared to preterm AGA

#### Shape Adjusted for BSA

Left ventricular volume was the major determinant of shape variance within the whole dataset in the unadjusted analysis. In the current study, BSA was a major determinant of LV volume (*R*^2^ = 0.53, *p* < 0.0001) and was therefore corrected for by adjusting left ventricular shape for BSA.

**Fig. 2:**
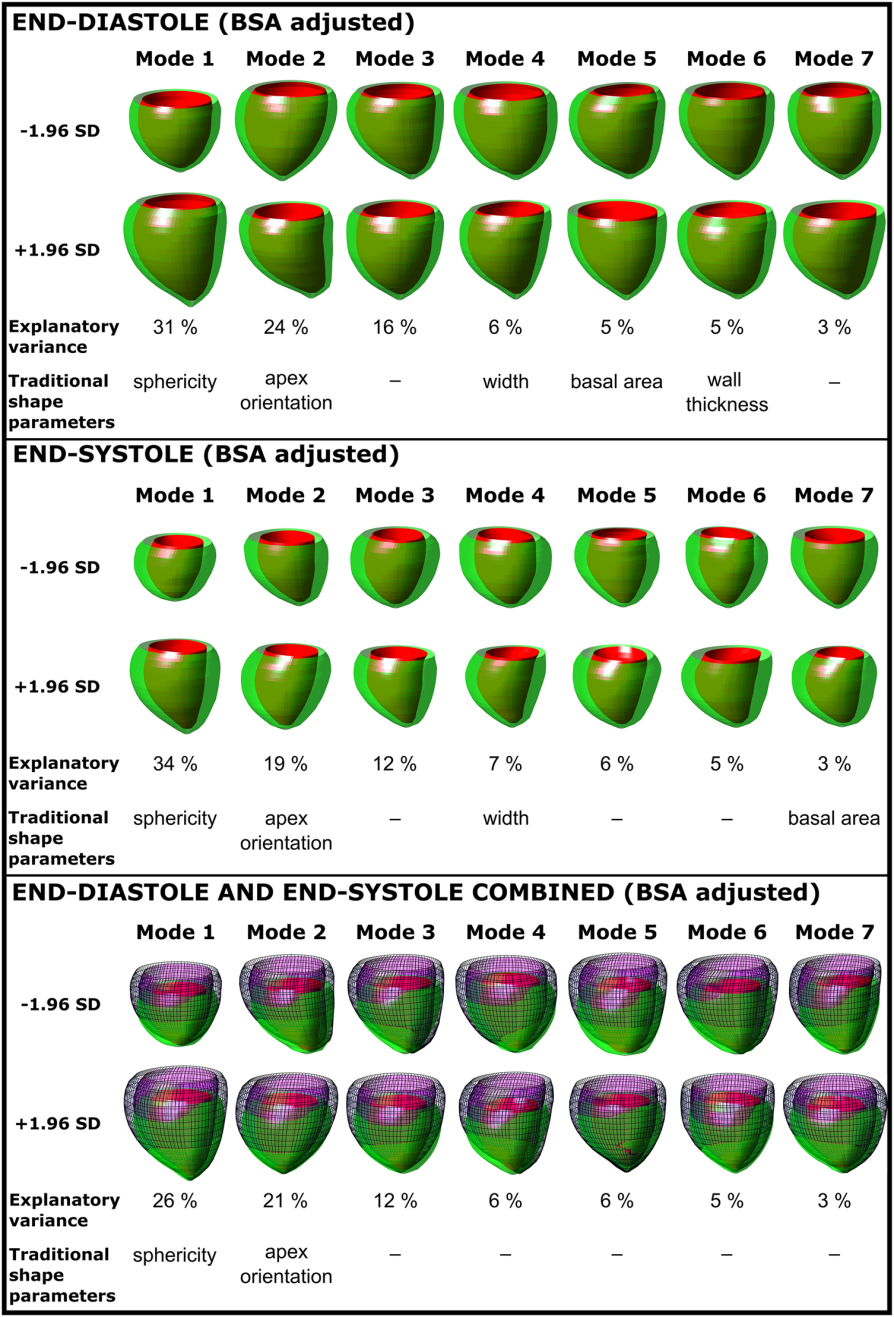
Modes 1–7 and their respective shape explanatory variance adjusted to body surface area (BSA) for end-diastole (top panel), end-systole (middle panel), and end-diastole and end-systole combined (bottom panel). 3D shapes shown are the ± 1.96 standard deviation extremes for each mode. The BSA-adjusted shape analysis captures the major explanatory variances while factoring in body surface area, a possible confounder in those born preterm or with fetal growth restriction

Figure 2 shows BSA-adjusted shape modes within the whole dataset for end-diastolic, end-systolic and the combination of both shapes. After adjustment for BSA, the first seven modes explained 89% of end-diastolic, 86% of end-systolic and 79% of combined end-diastolic and end-systolic shape variability. Sphericity (mode 1) explained 31%, 34%, and 26% of the variance of the end-diastolic, end-systolic, and combined shapes in the three groups.

**Fig. 3:**
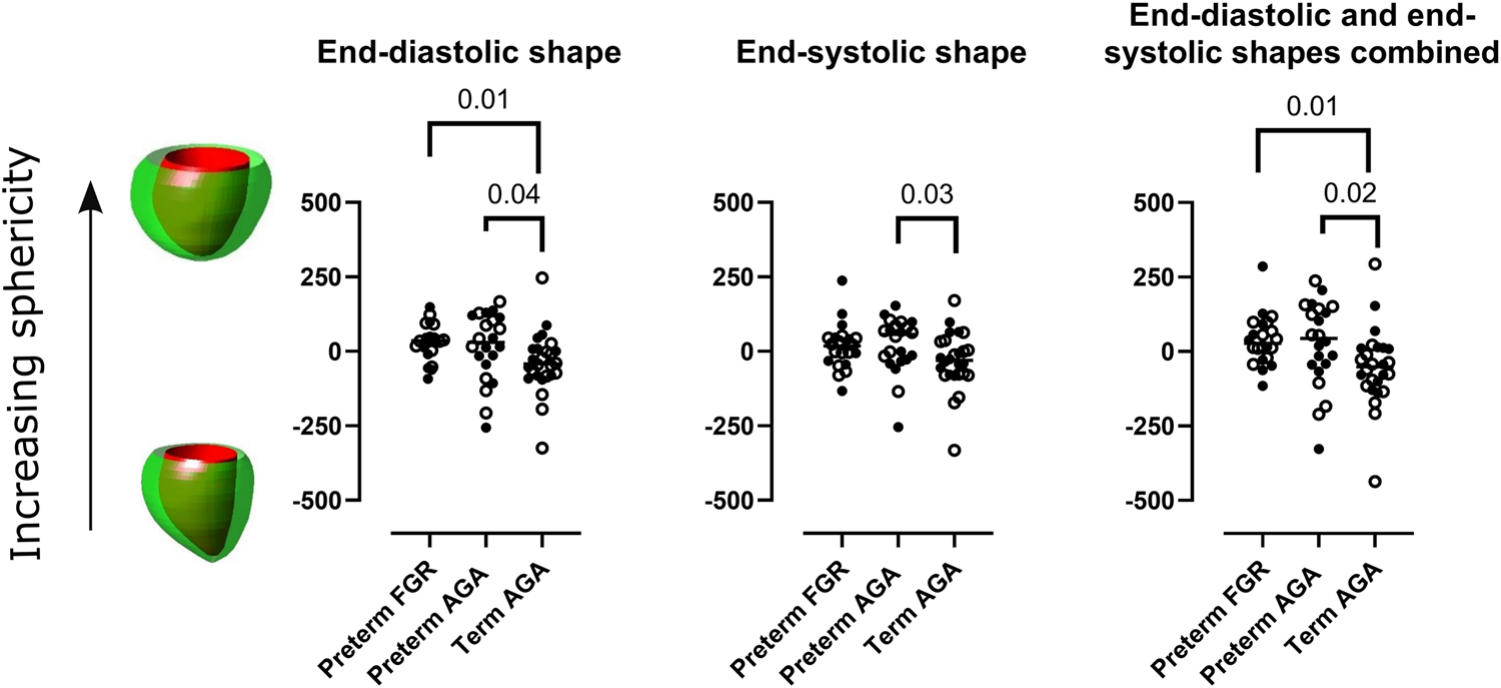
Sphericity explained the majority of shape variance in analysis adjusted to body surface area for end-diastole, end-systole, and end-diastole and end-systole combined. Both those born preterm after preceding fetal growth restriction (FGR) and those born preterm appropriate for gestational age (AGA) showed increased sphericity for end-diastole (left) and for end-diastole and end-systole combined (right) as compared to the term AGA group. The preterm AGA group also showed increased end-systolic sphericity as compared to the term AGA group (middle). Closed circles indicate boys, open circles indicate girls

Figure 3 and Table 3 show shape variability between the groups. For end-diastole, sphericity (mode 1) was higher in the preterm FGR group (− 35 vs 42; *p* = 0.02) and the preterm AGA group (− 30 vs 42); *p* = 0.04) as compared to the term AGA group. Basal area (mode 5) differed between the preterm AGA group (− 16 vs 15; *p* = 0.04) as compared to the term AGA group. For end-systole, sphericity (mode 1) was higher (− 57 vs 30; *p* = 0.03) for the preterm AGA group as compared to the term AGA group. Apex orientation (mode 3) differed between the preterm FGR group (− 15 vs 27; *p* = 0.04) as compared to the term AGA group. For the combined end-diastolic and end-systolic shape variability, sphericity (mode 1) was higher in the preterm FGR group − 27 vs 52; *p* = 0.01) and in the preterm AGA group (− 44 vs 52); *p* = 0.02) as compared to the term AGA group.

#### Shape Adjusted for EDV

**Fig. 4:**
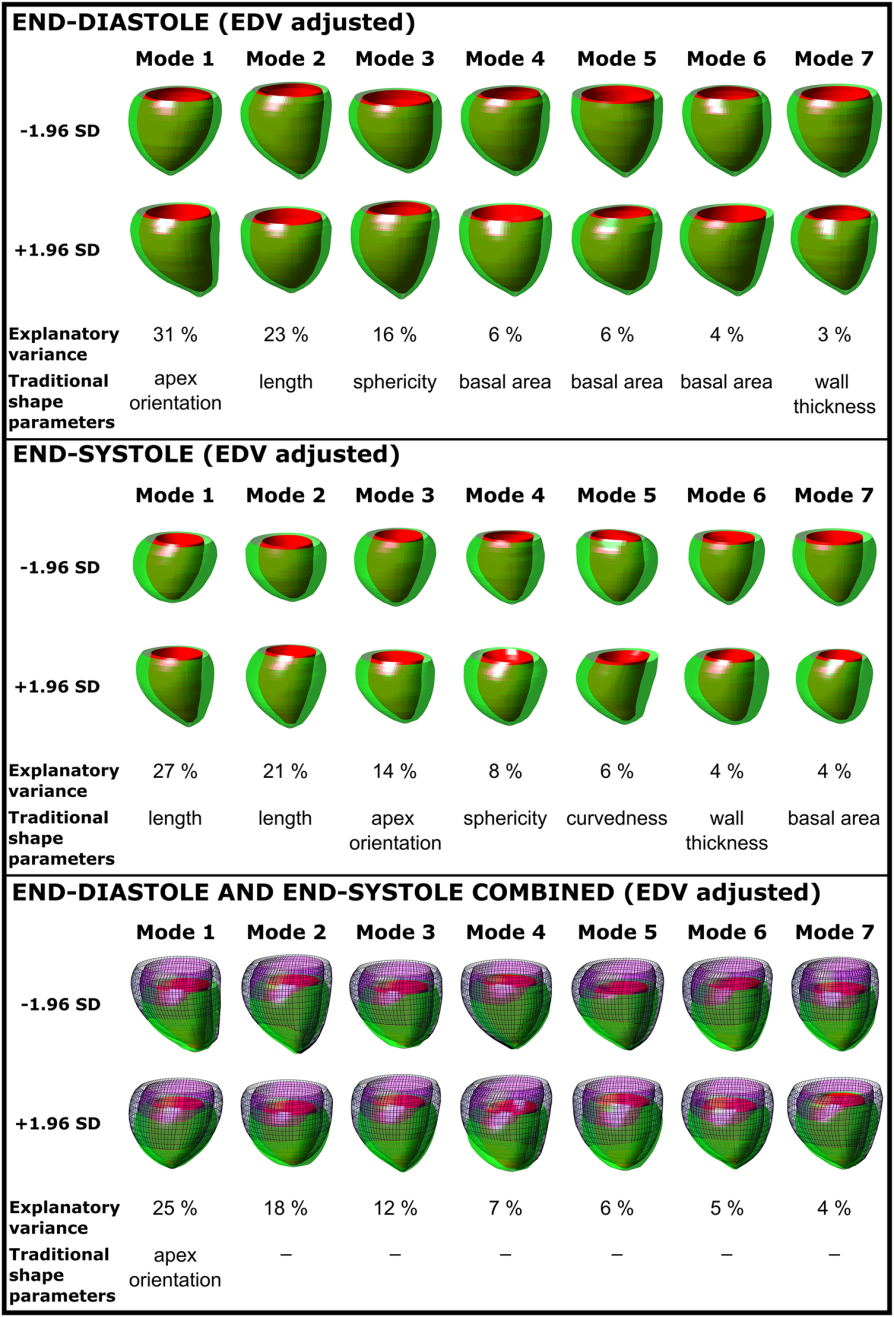
Modes 1–7 and their respective shape explanatory variance adjusted to end-diastolic volume (EDV) for end-diastole (top panel), end-systole (middle panel), and end-diastole and end-systole combined (bottom panel). 3D shapes shown are the ± 1.96 standard deviation extremes for each mode. The EDV-adjusted shape analysis captures the major explanatory variances while factoring in cardiac size, a possible confounder in those born preterm or with fetal growth restriction

To correct for LV volume, which was the main explanatory shape in the unadjusted shape analysis, the left ventricular shape was adjusted for left ventricular end-diastolic volume. After adjustment for end-diastolic volume, the first seven modes explained 87% of end-diastolic, 84% of end-systolic, and 77% of combined end-diastolic and end-systolic shape variability. Figure 4 shows EDV-adjusted shape modes within the whole dataset for end-diastole, end-systole, and the combination of both. For end-diastole, apex orientation (mode 1) explained 31%, LV length (mode 2) 23%, and sphericity (mode 3) 16% of the variance. For end-systole, LV length (modes 1 and 2) explained 48% of the variance. For the combined end-diastolic and end-systolic shape mode 1 explained: 25%, mode 2: 18% and mode 3: 12% of the variance.

Table 3 shows shape variability between groups for EDV-adjusted values. At end-diastole, sphericity was higher in the preterm FGR group (− 0.3 vs 0.5; *p* = 0.02) as compared to the term AGA group, whereas ventricular curvature (mode 5) differed between the preterm AGA group (0.3 vs − 0.3; *p* = 0.03). For the combined end-diastolic and end-systolic shape variability, mode 3 differed between the preterm AGA group (− 0.3 vs 0.8; *p* = 0.02) and the term AGA group, whereas mode 7 differed between the preterm FGR group (0.7 vs − 0.3; *p* = 0.02) and the term AGA group (no traditional shape parameter could be ascribed).

## Discussion

Adolescents born very preterm showed increased left ventricular sphericity without a concomitant decrease in left ventricular function. Very preterm birth preceded by early-onset FGR increased sphericity more so than very preterm birth in itself without additional alterations in cardiac function.

Adolescents born very preterm with birth weight appropriate for gestational age showed smaller LV volume as compared to those born at term with appropriate birth weight. A visual trend of smaller LV volume was observed also for the preterm FGR group. This suggests that very preterm birth in itself, and not verified early-onset FGR, is the main culprit for long-term changes in cardiac volumes. Earlier studies have shown that both children and adults born preterm with or without FGR have smaller cardiac volumes [17, 26]. However, in most studies, birth weight or birth weight deviation alone has been used to define FGR, leading to inclusion of cohorts with heterogeneous developmental characteristics. Further, in the current study, no group differences were observed in LVM or standard clinical measures of LV function. In line with these findings, Harris et al. [14] showed that adolescents born very preterm had smaller cardiac volumes without alterations in cardiac function. Modern peri- and neonatal care, similar as for the study population in the current study, was suggested to explain the lack of cardiac dysfunction in comparison to earlier studies.

In the current study, LV volume and LV length, were the main drivers of shape variance within groups for the unadjusted and EDV-adjusted shape analyses, respectively. These analyses thus confirmed the significant inter-group differences in LV volume as shown by left ventricular manual delineations, verifying the shape method. For the EDV-adjusted analysis at end-diastole, sphericity was higher in the preterm FGR group as compared to the preterm AGA group, although the explanatory variance for sphericity (mode 3) was only 16%. Shape analysis was thus able to detect this difference between groups even though the analysis was influenced by the larger explanatory variance in LV volume and/or LV length between groups, indicating method robustness.

Left ventricular volume and LV length did not explain shape variance between groups using the BSA-adjusted analysis. This indicates that the BSA-adjusted analysis successfully corrected for both LV volume and LV length and captured the actual shape differences present between groups. The effect of body metrics on shape are thus adjusted for. This is visualized as the LV volume and LV length are the major contributor to shape variance only in the unadjusted results. Importantly, for all three analyses (end-diastolic, end-systolic, and the combination of both time frames) the first and most important mode was related to sphericity indicating that sphericity is the main driver when the shape analysis is adjusted for BSA. Adjusting for BSA may thus be appropriate for assessing and comparing true shape variance between groups, especially in groups with discrepancies in height, as in the current study. When performing the BSA-adjusted shape analysis, sphericity was indeed higher in both preterm groups as compared to the term AGA group. A visual trend of increasing sphericity was observed from term AGA to preterm AGA to preterm FGR for end-diastolic shape. This is partly in line with previous studies observing increased sphericity in end-diastolic shape after FGR, although these studies mainly included late preterm and late-onset FGR and controls born near term [27–30].

In contrast to earlier studies that show increased sphericity [27, 31–34], the current study showed increased sphericity without signs of concomitant decrease in cardiac function. An explanation to the differences in cardiac function between previous and the current study may be that the current study population, with verified early-onset FGR, was surveyed and actively delivered at the first sign of adverse fetal distress. It could be hypothesized that the long-term effects of FGR might be decreased in this cohort, showing normal cardiac function, compared to earlier cohorts, who might have been subject to a compromised feto-placental circulation for a longer period. This hypothesis is strengthened by recent studies indicating similar findings with normal kidney function and only subtle sex-specific changes in arterial stiffness and blood pressure [12, 13]. Some [14, 35], but not all [26] recent studies show similar indications of less detrimental long-term effects on the cardiovascular system after preterm birth. Nevertheless, the increased sphericity and other shape differences found in the preterm groups in the current study could still be an early sign of structural remodeling. Although standard clinical measures of cardiac function were normal in the current study, alterations in cardiac shape, e.g., increased sphericity, have been linked to future decline in cardiac function [15].

This is the first study to present shape analysis of the combinatory end-diastolic and end-systolic shapes, displaying and analyzing the shape change between the two cardiac phases. The modes in the combined analysis were generally more difficult to categorize using the traditional shape parameters. However, in the BSA-adjusted analysis, mode 1 corresponded to sphericity (Fig. 2) and was, as in the end-diastolic analysis, increased for both preterm groups as compared to the term AGA group. Shape alterations and concomitant markers of cardiac dysfunction in FGR fetuses in utero have been shown to predict adverse cardiovascular outcomes at 6 months of age, establishing a possible link between shape differences and cardiac remodeling to a dysfunctional cardiovascular system [36]. Whether these changes found in childhood are permanent or rather a relatively acute and reversible change in cardiac function and structure, relative to the peri- and neonatal environment, is not known. In the current study, standard clinical measures of cardiac function, such as ejection fraction, left ventricular mass and stroke volume, were normal. The differences in the shape analysis shown in the current study, with increasing sphericity after preterm birth, also in the combined end-diastolic and end-systolic analysis, may thus indicate a change in pumping mechanics, possibly impacting future cardiac morphology and function [37], not yet visible using classical functional parameters.

In the current study, PCA was used to determine the modes best describing the left ventricular shape variance. Sphericity was shown to be one of the most explanatory variables in the BSA-adjusted analyses. Although values are not directly comparable to the left ventricular sphericity index, commonly assessed using echocardiography or CMR, both methods show the underlying relationship between ventricular width and length as a predictor of worsening long-term cardiovascular outcome [38, 39].

### Limitations

Not all study participants from the original study population were included in the current study, possibly increasing the risk for inclusion bias and a loss of statistical power. However, only limited peri- and neonatal differences were observed between those who agreed to participate and those who chose to drop out [13]. The strict inclusion criteria, including verified early-onset FGR and close matching between groups, also limits potential loss of statistical power due to loss to follow up.

## Conclusion

Adolescents born very preterm showed an increase in left ventricular sphericity. Sphericity was exacerbated by early-onset FGR, indicating an additive effect to that of very preterm birth on of left ventricular remodeling. Increased sphericity may potentially serve as a prognostic biomarker of future cardiovascular disease in this cohort, currently showing no signs of cardiac dysfunction using standard clinical measurements.
